# Comparative efficacy of combination of 1 L polyethylene glycol, castor oil and ascorbic acid versus 2 L polyethylene glycol plus castor oil versus 3 L polyethylene glycol for colon cleansing before colonoscopy: Study protocol of a randomized, double-blind, single-center study

**DOI:** 10.1097/MD.0000000000010481

**Published:** 2018-04-27

**Authors:** Xu Tian, Wei-Qing Chen, Xiao-Ling Liu, Hui Chen, Bang-Lun Liu, Yuan-Ping Pi

**Affiliations:** a Department of Gastroenterology, Chongqing Key Laboratory of Translational Research for Cancer Metastasis and Individualized Treatment, Chongqing University Cancer Hospital & Chongqing Cancer Institute & Chongqing Cancer Hospital, Chongqing 400030, China; b Editorial Office, TMR Integrative Nursing, TMR Publishing Group, Tianjin; c Department of Nursing, Chongqing University Cancer Hospital & Chongqing Cancer Institute & Chongqing Cancer Hospital, Chongqing, China.

**Keywords:** ascorbic acid, Boston Bowel Preparation Scale, bowel preparation, castor oil, colonoscopy, colorectal cancer, polyethylene glycol

## Abstract

Colonoscopy has been regarded as an important method of early diagnosing and treating gastrointestinal lesions; however adequate bowel preparation is critical one of many factors needed for successful colonoscopy. Although several modified or novel regimes have been developed, desired quality of bowel preparation has not yet been generated. Scattered evidences revealed that castor oil may have potential of effectively cleansing colon. It is noted that, however, prospective trial of exploring the value of castor oil in preparing bowel before colonoscopy is lacking. The aims of this study are to test the hypotheses that low dose castor oil (30 mL) may enhance potential of polyethylene glycol (PEG) and combination of low castor oil and ascorbic acid may halve the volume of PEG.

This is a randomized, double-blind (endoscopist and assessor), single center trial with three-arm design. We will randomly assign 282 adult patients (≥18 years but < 75 years), who are scheduled to undergo colonoscopy, to receive either 3 L PEG alone, 2 L PEG plus 30 mL castor oil or combination of 1 L PEG, 30 mL castor oil and 5 g ascorbic acid. The bowel preparation quality based on Boston Bowel Preparation Scale (BBPS) is the primary outcome. The secondary outcomes include the first defecation time, total number of defecation, time of cecal intubation, detection rate of polyp and adenoma, willing to repeat the same regime, tolerance to regime, and adverse events.

The study protocol has been approved by the Clinical Research Ethics Committees of Chongqing University Cancer Hospital & Chongqing Cancer Institute & Chongqing Cancer Hospital & Chongqing Cancer Center (2017[107]). The results from this trial will be submitted for publication in peer-reviewed journals, and will be presented at national and international conferences.

## 1 Introduction

Colorectal cancer (CRC) remains one of the most prevalent cancers in both sexes worldwide^[1]^ and is also the critical contributor to high cancer-related morbidity and mortality.^[2]^ Colonoscopy has been regarded as the important method of early detecting CRC and effectively treating gastrointestinal lesions.^[3]^ Evidences suggest that the mortality of CRC will be approximately reduced by 50% if polyps and abnormal lesions in digestive tract were early detecting and endoscopic resected.^[4,5]^ However, adequate bowel preparation is the prerequisite of effective and safe colonoscopy.^[6]^ Some studies reported that inadequate bowel cleansing is responsible for more than 40% of colonoscopy failures.^[7]^ Moreover, poor bowel preparation will also decrease the detection rate of polyp and adenoma,^[8]^ prolong the operation time,^[9]^ and increase the risk of procedure related complications and medical expenditure.^[10]^

In everyday practice, multiple factors will impair quality of colon cleans.^[11]^ Of those all factors, low patient compliance to, poor palatability of and required liquid of preparation solution account for 20% to 25% of inadequate bowel preparations.^[6]^ To date, a large number of modified or novel bowel preparation regimes such as split dose polyethylene glycol (PEG) and low-dose PEG plus ascorbic acid have been developed in order to obtain adequate bowel preparation.^[3]^ However, desired bowel preparation has not yet been obtained. It remains a changeling to optimize the bowel preparation efficacy prior to colonoscopy.

Castor oil is a safe, effective, and cheap laxative with highly efficacious for colon cleans,^[12]^ which has been widely used as the laxative in many settings such as intravenous urography.^[13–15]^ Apisarnthanarak et al^[12]^ found that castor oil and sodium phosphate (NaP) obtained similar patients’ satisfaction and efficacy of colon cleans for barium enema. The trial performed by Yang et al^[15]^ observed no difference in laxative efficacy between castor oil and bisacodyl for intravenous urography. It must be noted that, moreover, combination of bisacodyl and PEG^[16]^ and combination of NaP^[17]^ obtained promising bowel preparation efficacy, decreased the required volume of liquid, and improved the compliance with the recommended regime when compared to standard PEG regime. Moreover, the potential of 2 L PEG plus ascorbic acid is equal to that of 3 L PEG,^[18]^ and which is better than that of 2 L PEG plus NaP.^[19]^ And thus, we speculate that combination of 2 L PEG and castor oil may have similar potential with 2 L PEG plus bisacodyl or NaP for colon cleanses, and which is not inferior to the potential of 3 L PEG. More importantly, 2 trials^[20,21]^ found that combination of 1 L PEG, bisacodyl, and ascorbic acid improved the patient's tolerability and did not compromise bowel preparation efficacy compared with combination of 2 L PEG and ascorbic acid. So, we also speculate that combination of castor oil and ascorbic acid may have the potential of halving the required volume of PEG.

Published evidences^[22–24]^ suggested that large dose castor oil (50 or 60 mL) may be more likely to cause some adverse effects such as abdominal cramping, vomiting, nausea, abdominal fullness, fainting, and insomnia. However, some trial^[12]^ found that the incidence of adverse events in low dose castor oil (30 mL) is lower than that of NaP. So, we hypothesize that 30 mL castor oil may enhance potential of PEG, and combination of 30 mL castor oil and ascorbic acid (5 g)^[25]^ may halve the required volume volume of PEG.

## 2 Methods and analysis

We developed this protocol according to the Recommendations for Interventional Trials (SPIRIT).^[26]^ The trial is registered at Chinese Clinical Trial Registry (www.chictr.org.cn) with identifier ChiCTR-IIR-17012418.

### 2.1 Objectives

The aims of this trial are to investigate the potential of 30 mL castor oil in enhancing colon cleans and the role of combination of 30 mL castor oil and 5 g ascorbic acid in halving the required volume of PEG.

### 2.2 Trial design and setting

**Figure 1:**
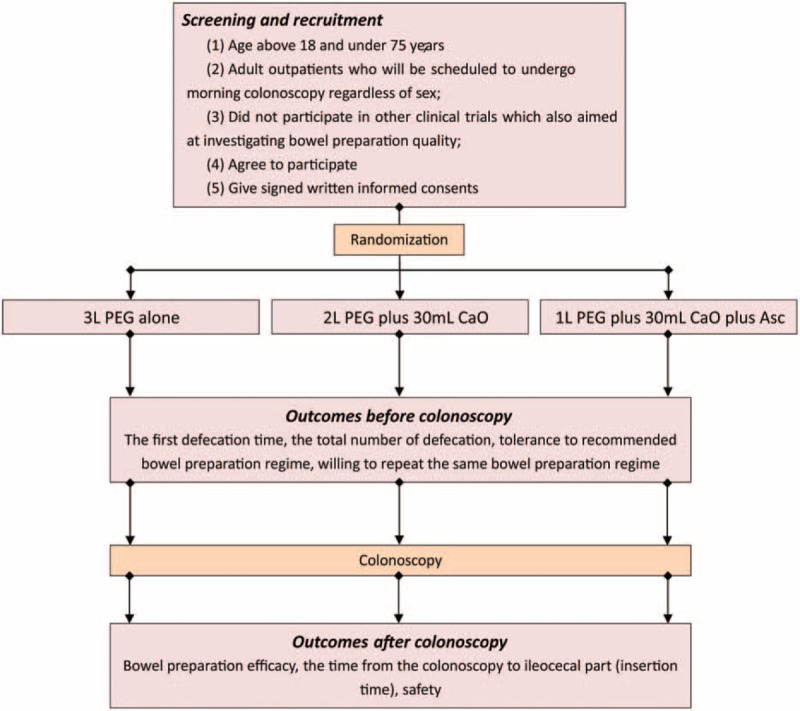
Flowchart of the study. Asc = ascorbic acid, CaO = castor oil, PEG = polyethylene glycol.

This randomized, double-blind (endoscopist and assessor), single center trial with three-arm is designed to explore the potential of 30 mL castor oil in preparing bowel prior to colonoscopy. We will enroll and randomly assign all eligible candidates to receive either 3 L PEG, combination of 30 mL castor oil, and 2 L PEG or combination of 30 mL castor oil, 5 g ascorbic acid and 1 L PEG (see Fig. 1). This study will be conducted in the Departments of Gastroenterology of Chongqing University Cancer Hospital & Chongqing Cancer Institute & Chongqing Cancer Hospital in Mainland China.

### 2.3 Participant selection

#### 2.3.1 Inclusion criteria

Participants will be enrolled if the following criteria were met: age above 18 and under 75 years; adult outpatients who will be scheduled to undergo morning colonoscopy regardless of sex; did not participate in other clinical trials which also aimed at investigating bowel preparation efficacy; and agree to participate, and give signed written informed consents.

#### 2.3.2 Exclusion criteria

We will exclude those patients if any of the following criteria were met: lactation; pregnancy; experienced the abdominal surgery such as gynecogic surgery, appendectomy, and laparoscopy; neurological diseases; contraindication of colonoscopy, allergy to ingredients of PEG, castor oil or ascorbic acid or medically vulnerable populations (decompensated liver disease, chronic heart failure, known renal diseases or glomerular filtration rate [GFR] < 30), socially vulnerable populations such as prisoners and incapable of consent, patients with high risk for preparation failure such as narcotic use, chronic constipation, past inadequate preparation, and known gastrointestinal motility disorders.

#### 2.3.3 Sample size calculation

The bowel preparation efficacy will be primarily tested, and thus we will calculate the anticipated sample size based on this given outcome. According to the findings from the previous studies,^[18,20]^ we propose that the rate of adequate bowel preparation in 3 L PEG, combination of 2 L PEG and 30 mL castor oil, and combination of 1 L PEG, 30 mL castor oil and 5 g ascorbic acid will be 85%, 90%, and 95%. We assumed the significance and power to be 0.05% and 80%, respectively, and thus the sample size required to detect difference will be 255 patients. Because the dropout rate is expected to be 10%, totally 282 patients should be enrolled.

#### 2.3.4 Recruitment, randomization, and blinding

The day before colonoscopy, investigators who have been trained for the study and are authorized by the principal investigator will assess candidates according to the inclusion and exclusion criteria. After eligible patients were identified, the written informed consents will be obtained from the eligible patients, their next of kin or legal representatives. At the phase, the investigators will also collect demographic and clinical characteristics data of all eligible patients, which includes sex, age, body weight, body mass index, reasons for colonoscopy, previous colonoscopy history, and comorbidity such as hypertension, diabetes mellitus, and cardiovascular disease.

All recruited patients will be randomly assigned to receive one of the 3 preparation regimes including 3 L PEG, combination of 2 L PEG and 30 mL castor oil, and combination of 1 L PEG, 30 mL castor oil, and 5 g ascorbic acid. We will generate the random sequence by using SPSS 17.0 software, and the random sequence will be sealed in opaque envelope. An independent research nurse will be designated to random all patients into 3 different groups according to the random sequence.

In order to eliminate the risk of bias as much as possible, the endoscopists will be blinded except the research nurse who conducted the randomization procedure during examine period. Moreover, we will also blind the biostatistician.

#### 2.3.5 Study protocol

According to the findings from our previous meta-analysis,^[27]^ all participants enrolled in the study will be instructed to take low fat and residue diet without food color the day before colonoscopy, and all begin to fast at 20:00 pm on the day before colonoscopy. Patients can allow eating bun, bread, and chocolate in order to enhance tolerance, decrease incidence of adverse events such as hypoglycemia and cardio-cerebral vascular accidents if they experienced serious hunger feeling.

**Figure 2:**
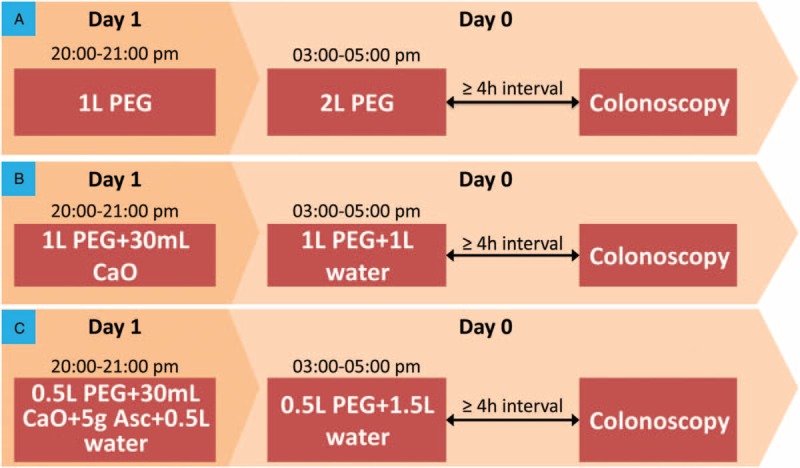
Study protocol for each group. The protocol for 3 L PEG alone group was depicted in (A) protocol for 2 L PEG plus 30 mL castor oil was displayed in (B), and protocol for combination of 1 L PEG, 30 mL and 5 g ascorbic acid was delineated in (C). PEG = polyethylene glycol.

The study protocols of all 3 groups were graphically depicted in Figure 2. According to the recommendation from US Multi-Society Task Force on Colorectal Cancer,^[28]^ we will instruct all patients to ingest bowel preparation regime with split-dose. So, the 3 L PEG alone group will be instructed to ingest 1 L of PEG at 20:00 to 21:00 PM on the day before colonoscopy and the remaining 2 L at the 3:00 to 5:00 AM on the morning before colonoscopy (Fig. 2A). The combination of 2 L and 30 mL castor oil group will be instructed to ingest 1 L of PEG and 30 mL castor oil at 20:00 to 21:00 PM on the day prior to colonoscopy and the remaining 1 L of PEG and extra 1 L clean water at the 3:00 to 5:00 AM on the morning before colonoscopy (Fig. 2B). The combination 1 L PEG, 30 mL castor oil and 5 g ascorbic acid group will be instructed to ingest 0.5 L of PEG, 30 mL castor oil and 5 g ascorbic acid and extra 0.5 L clean water at 20:00 to 21:00 PM on the day before colonoscopy and the remaining 0.5 L of PEG and extra 1.5 L clean water at the 3:00 to 5:00 AM on the morning before colonoscopy (Fig. 2C). The patients were instructed to digest PEG 250 mL every 15 minutes. Moreover, all eligible participants will be admitted to take oral 10 mL dyclonlne and 20 mL clean water at the 10 minutes prior to colonoscopy. For all eligible patients, propofol injection will be intravenously administered for sedation.

#### 2.3.6 Rescue therapy

For patients with inadequate bowel preparation, direct investigators will judge whether the colonoscopy should be continued or discontinued. If the inadequate bowel preparation is not suitable for colonoscopy, patients will be instructed to prepare bowel again using the regimes freely provide by research group. Moreover, if serious side effects occur, investigators will provide quick and appropriate treatment (including drug administration), based on their medical judgement. All treatment or drug administration will be recorded.

#### 2.3.7 Study endpoints

##### 2.3.7.1 Primary outcome

The bowel preparation efficacy was regarded as the primary outcome, which will be assessed by using the Boston Bowel Preparation Scale (BBPS). As one of the commonest scales of assessing quality of bowel preparation,^[29]^ the BBPS evaluates right (including the cecum and ascending colon), transverse (including the hepatic and splenic flexures), and left (including the descending colon, sigmoid colon, and rectum) colon on a 4-point scoring system with a total score out of 9.^[30]^ In this scoring system, a score of 9 was summed if the whole colon was perfectly cleaned without any residual liquid, and a score of 0 was calculated if colonoscopy is impossible.^[31]^ The adequate bowel preparation was defined as having total score > 5 and no segment < 2 in the present study.^[32]^ The direct endoscopists will be trained to use the BBPS to grade the quality of bowel preparation during colonoscopy.

##### 2.3.7.2 Secondary outcomes

We will also measure the first defecation time (the endoscopists record the time of first defecation after digesting regime), the total number of defecation (the direct investigators record the number of defecation before performing the colonoscopy), the time of cecal intubation (the endoscopists record the time of starting procedure to reaching ileocecal part), detection rate of polyp and adenoma (the number of polyp and adenoma during colonoscopy), tolerance to recommended regime (the research nurse ask the patients answering the questioner with a 4-point scale), and willing to repeat the same bowel regime (the research nurse instruct the patients to answer whether they will select the same regime to perform the bowel preparation) as the secondary outcomes.

##### 2.3.7.3 Safety assessments

The direct investigator will record all adverse events related to bowel preparation regime and colonoscopy such as nausea, vomiting, bloating, abdominal pain, and electrolyte imbalance into the case report form (CRF). That is to say, when an adverse event occurs, the investigator will record the symptoms and signs of the adverse reaction, the duration (start and end date), severity, course, outcome, significance, and any action taken in relation to the adverse event. It is noted that any symptom that existed before the start of the bowel preparation will not be recorded as adverse events.

##### 2.3.7.4 Laboratory tests

The 2 mL blood sample will be collected before and after the bowel preparation. These all samples will be transferred to clinical laboratory of Chongqing University Cancer Hospital & Chongqing Cancer Institute & Chongqing Cancer Hospital in order to know and further compare the change of all electrolytes such as serum sodium and potassium. All blood samples will be probably handled after analyzed in accordance with Chinese guidelines of Good Clinical Practice (GCP).

##### 2.3.7.5 Data management

All original data will be recorded in the CRF severally. The completed CRF, after signed by the direct investigators, will be transferred to the head investigator (W-QC). Data entry will be doubly performed by 2 investigators using the Excel 2010. Head investigator and research nurse who is responsible for data collection, sort, and dataset construction have the right of accessing to the final trial dataset. More importantly, all process associated with data access and analysis will be supervised by the Clinical Research Ethics Committees of Chongqing University Cancer Hospital & Chongqing Cancer Institute & Chongqing Cancer Hospital.

##### 2.3.7.6 Statistical analysis

In this study, we will adopt intent-to-treat (ITT) and per-protocol (PP) method to analyze all data. Demographic and clinical characteristics will be summarized with means, medians, and standard deviations. To assess differences between groups, data will be analyzed with Mann–Whitney *U*-test, Student's *t* test and Chi-square test according to the variables evaluated. Differences between groups regarding the bowel preparation efficacy will be analyzed with the Chi-square test. We will perform the subgroup analyses according to the indication for colonoscopy and previous history of colonoscopy. It is noted that all statistical analyses will be conducted with 2-tailed test, and *P* values of < .05 will be considered as considered significant. All statistical analyses will be performed by blinded professional statisticians with SPSS ver. 17.0 for Windows (SPSS Inc., Chicago, IL).

## 3 Discussion

Although several advanced treatments for CRC have been developed, CRC remains one of the common cancers worldwide.^[1]^ Although many noninvasive tests such as fecal immunochemical test, fecal occult blood test, and CT colonography are available,^[33]^ colonoscopy has been used a preferred method to early diagnosing CRC and treating gastrointestinal lesions,^[3]^ and the issued data suggested that the colonoscopy was associated with the decreased mortality of CRC.^[27]^ It is noted that, however, the inadequate bowel preparation will significantly decrease the effectiveness and safety of this given procedure.^[6]^ A numerous of regimes such as PEG, NaP, and sodium picosulphate with magnesium citrate have been developed to clean colon before colonoscopy,^[34]^ but the effectiveness of all these regimes are not still satisfactory.^[35]^ And thus, it is essential to develop novel bowel preparation regime.

Some published evidences suggested that 30 mL castor oil has the potential of enhancing colon cleans compared to NaP.^[12]^ Moreover, evidence also demonstrated similar laxative efficacy between castor oil and bisacodyl.^[15]^ However, it is unclear if 30 mL castor oil may enhance the potential of PEG prior to colonoscopy.

So we design the randomized, double-blind, single center with three-arm design to test whether 30 mL castor oil may enhance the potential of 2 L PEG and combination of 30 mL and 5 g ascorbic acid may halve the required volume of PEG. The results of the trial will influence evidence based decision making for bowel preparation regimen prescriptions as it will be fundamental in providing reliable recommendations for bowel preparation regimens before colonoscopy.

## 4 Ethics and dissemination

The study protocol has been approved by the Clinical Research Ethics Committee of the Chongqing University Cancer Hospital & Chongqing Cancer Institute & Chongqing Cancer (2017[107]). In order to guarantee the right of all eligible patients during the study period, we will strictly follow the Declaration of Helsinki and Chinese guidelines of Good Clinical Practice.

During the screening and recruitment, the investigators must explain to each eligible candidate in detail the purposes, procedures, and potential benefits and risks of the study. At the same time, the investigators must also let every potential patient know that he/she has the right to withdraw consent at any time during the study period. We will give sufficient time to each potential patient so that the decision of participating in the clinical trial can be rationally made. Every patient or the authorized surrogate of the patient must give the written informed consent before considered for enrolment in the study. All written informed consents will be kept as a part of the clinical trial documents.

All investigators will be informed that personal information provided by every patient will only be used to research. These all information will not be used to achieve other purposes which are not anticipated in the study. Certainly, all process will be performed according to the Chinese guidelines of Good Clinical Practice. Results of the study will be submitted to peer-reviewed academic journals, and will be presented at national and international conferences.

## Author contributions

**Conceptualization:** Xu Tian, Wei-Qing Chen, Yuan-Ping Pi.

**Data curation:** Xu Tian, Xiao-Ling Liu, Bang-Lun Liu, Yuan-Ping Pi.

**Formal analysis:** Xu Tian, Bang-Lun Liu.

**Investigation:** Xu Tian, Xiao-Ling Liu, Hui Chen.

**Methodology:** Xu Tian, Wei-Qing Chen.

**Resources:** Xu Tian, Hui Chen.

**Software:** Xu Tian, Bang-Lun Liu.

**Validation:** Xu Tian.

**Writing – original draft:** Xu Tian.

**Writing – review & editing:** Wei-Qing Chen, Yuan-Ping Pi.
